# Does the Duration of Each Waldenström Stage Affect the Final Outcome of Legg–Calvé–Perthes Disease Onset before 6 Years of Age?

**DOI:** 10.3390/children8020118

**Published:** 2021-02-06

**Authors:** Ho-Seok Oh, Myung-Jin Sung, Young-Min Lee, Sungmin Kim, Sung-Taek Jung

**Affiliations:** Department of Orthopedic Surgery, National University Hospital, Gwangju 61469, Korea; koreankid07@naver.com (H.-S.O.); smj2383@naver.com (M.-J.S.); lovepoplove@naver.com (Y.-M.L.); kimsum83@gmail.com (S.K.)

**Keywords:** Legg–Calvé–Perthes disease, Herring lateral pillar classification, Stulberg classification, Waldenström stage, duration

## Abstract

The purpose of this study was to evaluate the outcomes of patients with Legg–Calvé–Perthes disease (LCPD) with disease onset before 6 years of age who were treated with conservative methods and to identify prognostic factors. Moreover, we evaluated the duration of the Waldenström stage and its correlation with the disease outcome. Disease severity was assessed using the lateral pillar classification, and the final outcome was evaluated using the Stulberg classification. We divided patients with LCPD into two groups according to the age at onset: group 1 (<4 years) and group 2 (4–6 years). The final outcomes of the two groups were compared. We also assessed the duration of each Waldenström stage. LCPD was noted in 49 hips of 49 patients. The lateral pillar class was A in one patient, B in 29 patients, and B/C or C in 19 patients. The Stulberg class was I or II (good) in 30 patients, III (fair) in 13 patients, and IV or V (poor) in six patients. The lateral pillar class significantly correlated with the final outcome. Groups 1 and 2 comprised 25 and 24 patients, respectively. The prevalence of good outcomes did not significantly differ between the groups (*p* = 0.162). The duration of the initial stage was 4.1 months in the good outcome group and 6.2 months in the fair or poor outcome group. The duration of the fragmentation stage of the femoral head was 5.9 months in the good outcome group and 11.9 months in the fair or poor outcome group. The durations of initial and fragmentation stages significantly differed between good outcome group and fair or poor outcome group (*p* = 0.009 and *p* < 0.001, respectively). The prognosis of patients with disease onset before the age of 6 years was favorable. The disease severity and duration of each Waldenström stage can be predictors of the outcome. Patients with prolonged initial and fragmentation stages showed worse outcomes and often required more active treatment to shorten the durations of the initial and fragmentation stages.

## 1. Introduction

The prognosis of Legg–Calvé–Perthes disease (LCPD) varies with the patient’s age at disease onset. LCPD occurring in children under 6 years of age is usually a benign, self-limiting condition with a good outcome [1,2,3]. However, extensive femoral head involvement in patients with early onset is associated with a potentially poor outcome. In children under 6 years of age, the Catterall and lateral pillar classes of LCPD correlate with the prognosis [4,5,6,7].

The Waldenström classification of LCPD is based on radiographic changes over time as the disease progresses naturally. According to the classification, LCPD has four radiographic stages: initial, fragmentation, reossification, and residual [8]. In a retrospective study, the time from the first radiographic evidence of disease to the start of fragmentation was a mean of 6 (range: 1–14) months, with fragmentation and reossification stages lasting 8 (range: 2–35) and 51 (2–122) months, respectively. The disease severity positively correlated with the duration of each stage, particularly the healing stage [9]. However, few studies have attempted to correlate the duration of each stage with the outcome in younger patients [9,10,11,12].

The purpose of this study was to clarify the outcome of patients with LCPD with disease onset before 6 years of age who were treated with conservative methods, and to identify the prognostic factors, such as the age of disease onset and disease severity. Moreover, we evaluated the duration of each Waldenström stage and correlated it with the outcome.

## 2. Materials and Methods

Of a total of 230 hips of patients with LCPD diagnosed before 6 years of age, treated at the Chonnam National University Hospital from 1980 to 2015, 66 with adequate radiographs at presentation showed the initial stage and were followed-up until skeletal maturity. We excluded patients who were treated surgically (*n* = 14) or considered to have completely bypassed fragmentation stage (*n* = 3). Finally, we included 49 hips of 49 patients.

**Figure 1:**
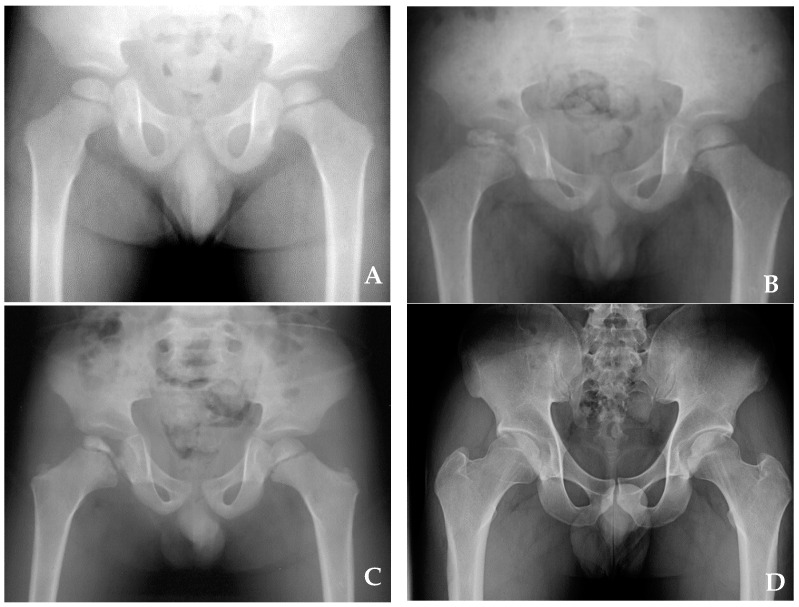
Anteroposterior radiograph of patients at the age of 2 years. Initial (**A**), fragmentation (**B**), reossification (**C**), and residual (**D**) stages.

There were 47 boys and two girls. The patients’ mean age at diagnosis was 3.9 (range: 1.9–5.9) years, and the mean follow-up duration was 14.3 (range: 6.2–27.8) years (Table 1). The medical records of all patients were reviewed to extract data on the sex, age of onset, treatment method, and follow-up duration. Moreover, two orthopedic surgeons (HSO and SK) reviewed the standard anteroposterior and frog-leg radiographs of the hips throughout the treatment course (Figure 1). To evaluate disease severity, we applied the lateral pillar classification when the disease showed maximal epiphyseal fragmentation in the fragmentation stage [12,13].

To determine the Waldenström class, the interval between the first radiograph showing features of one stage and the first radiograph showing features of the next stage was determined as the duration of the stage. We used the modified Waldenström classification published by Joseph et al. [14] to determine the onset of initial, fragmentation, and reossification stages. The onset of the initial stage was defined as the time when part or whole of the epiphysis was sclerotic (Figure 1A). When one or two vertical fissures were present in the anteroposterior or frog-leg lateral view, we marked the onset of the fragmentation stage (Figure 1B). The onset of the reossification stage was defined as the time when early new bone was visible lateral to the fragmented epiphysis (Figure 1C).

The final outcome was evaluated radiographically using the Stulberg classification. The Stulberg class was determined from radiographs at the time of skeletal maturity, and patients without skeletal maturity at the final follow-up were excluded (Figure 1D) [13]. Stulberg classes I and II were considered to be good, III to be fair, and IV and V to be poor.

To evaluate whether or not age is a factor determining the prognosis of patients with LCPD before 6 years of age, we divided the patients into two groups according to the age at disease onset: group 1, <4 years of age, comprising 25 hips; and group 2, 4–6 years of age, comprising 24 hips. Both groups were compared using the Stulberg classification.

Outcomes were analyzed in terms of disease severity and age difference, with Fisher’s exact probability test, using IBM SPSS statistics 21.0. Differences in the duration of each stage between the good and fair or poor outcome groups were analyzed with independent *t*-tests. A *p*-value < 0.05 was considered to be statistically significant. The kappa statistic was computed to test the inter-rater reliability of the Waldenström, lateral pillar, and Stulberg classifications. Cohen’s kappa values of 0.61–0.80 were interpreted as substantial agreement, whereas values of 0.81–1.00 were interpreted as almost perfect agreement [14].

## 3. Results

Patients diagnosed with LCPD under the age of 6 years were classified using Herring’s lateral pillar classification at the time of maximum fragmentation. Only one hip was classified as lateral pillar A and showed a good outcome (Stulberg I or II). Of 29 hips classified as lateral pillar B, 26 showed a good outcome, whereas three showed a fair outcome (Stulberg III). Of 19 hips classified as lateral pillar C, three, 10, and six showed good, fair, and poor outcomes, respectively (Stulberg IV or V; Table 2). The lateral pillar class significantly correlated with the final outcome (*p* < 0.001). Regarding agreement using Cohen’s kappa, almost perfect agreement was obtained for the Herring’s lateral pillar and Stulberg classifications (Cohen’s kappa of 0.85 and 0.83, respectively).

Of 25 hips in group 1, 14 showed a good outcome, whereas 11 showed a fair or poor outcome. Of 24 hips in group 2, 18 showed a good outcome, whereas six showed a fair or poor outcome. There were no significant differences between the age groups in terms of the final outcome (*p* = 0.162; Table 3).

The duration of the initial stage was 4.1 months (0.7~9.8) in the good outcome group and 6.2 months (2.6~13.5) in the fair or poor outcome group. The duration of the fragmentation stage was 5.9 months (1.0~16.0) in the good outcome group and 11.9 months (4.2~23.6) in the fair or poor outcome group. The durations of both initial and fragmentation stages significantly differed between the two groups (*p* = 0.009 and *p* < 0.001, respectively; Table 4). Regarding agreement using Cohen’s kappa, substantial agreement was obtained for the Waldenström classification (Cohen’s kappa of 0.77).

## 4. Discussion

In recent studies, LCPD diagnosed in children less than 6 years of age has shown good final outcomes. In studies by Rosenfeld et al. [6], Gent et al. [5], and Nakamura et al. [7], 80% (131/164), 65% (45/69), and 63% (72/114) of hips showed a good final outcome (Table 5), respectively. Moreover, all these studies revealed a significant correlation between the lateral pillar class and the final outcome. Although we only included patients with skeletal maturity at the final follow-up, excluding those with complete bypass surgery, the results of our study (61.2%, 30/49 hips) were consistent with those of other studies and showed a significant correlation between the lateral pillar class and the final outcome (Table 2). These data support the idea that the overall outcome of patients diagnosed with LCPD under 6 years of age is favorable.

Many studies have suggested that the final outcome of LCPD is significantly affected by patient age at the time of disease onset. The younger the patient at onset, the milder the disease severity [1,15,16,17]. Presumably, this is related to one or more of the following factors: a smaller volume of infracted bone, a more abundant circulation to the proximal femoral epiphysis, and an increased ability of bone to remodel in very young children [18]. A few studies have evaluated the correlation between age and the prognosis of patients diagnosed with LCPD under 6 years of age. Rosenfeld et al. reported [6] that the combination of young age (0 to 3 years 11 months) with lateral pillar class A or B significantly correlated with a better outcome in patients diagnosed with LCPD under 6 years of age. However, Nakamura et al. reported [7] that a good outcome did not significantly differ between younger (0 to 3 years 11 months) and older groups (4 years to 5 years 11 months). In our study, in patients diagnosed with LCPD under 6 years of age treated with conservative methods, the final outcome did not significantly differ between groups 1 and 2 (Table 4).

Waldenström classified LCPD based on radiographic changes into the initial, fragmentation, reossification, and residual stages [8], and the duration of each stage varied across patients. Benjamin Joseph et al. [10] reviewed 610 patients with LCPD and divided the disease progression into seven stages (Ia, Ib, IIa, IIb, IIa, IIIb, and IV) using the modified Elizabethtown classification, which subdivides Waldenström stages. The median durations of the initial (Ia and Ib), fragmentation (IIa and IIb), and reossification (IIIa and IIIb) stages were approximately 7, 8, and 18 months, respectively. However, the duration of any disease stage did not differ significantly among the Catterall groups in their study. Herring et al. [9] also reported that the duration of the fragmentation stage of the disease was approximately 9 months. Catterall et al. [11] suggested that the disease duration varies with the final outcome, with the disease duration being 8 months shorter in children with good results compared to those with poor results. In our study, despite only including patients with LCPD onset at less than 6 years of age, the median durations of the initial and fragmentation stages were approximately 5 and 8 months, respectively, showing a shorter duration of the initial stage and similar duration of the fragmentation stage compared to other studies. The duration of the initial stage positively correlates with that of the fragmentation stage. Moreover, prolonged initial and fragmentation stages are associated with a worse prognosis. Several events occur during the fragmentation period, including subluxation of the femoral epiphysis and collapse of the lateral pillar, that increase the risk for permanent femoral head deformity [10,13]. Accordingly, the duration of the initial stage positively correlates with that of the fragmentation stage, and in such cases, even if patients with LCPD are under 6 years of age, they may require active treatment such as surgical treatment.

This study has several limitations. First, we used several subjective staging systems. Although two orthopedic surgeons reviewed the radiographs to reduce bias, the subjectivity of the staging systems could have caused a bias. Second, features that characterized each stage may have preceded the date of each radiograph, resulting in overestimation of the stage duration. Third, the number of patients included in this study was too small for an optimal statistical analysis.

## 5. Conclusions

In conclusion, the prognosis of patients with LCPD onset before the age of 6 years treated with conservative methods is favorable. The durations of initial and fragmentation stages can predict the outcome; in particular, prolongation of the fragmentation stage can adversely affect the prognosis. Patients with a prolonged initial stage may require active treatment to shorten the durations of the initial and fragmentation stages.

## Figures and Tables

**Table 1:** Patient demographics.

Sex	Boy:Girl (Hips)	47:2
Location	Right:Left (hips)	24:25
Age	Average (Minimum~Maximum)	3.9 (1.9~5.9)
Follow-up duration	Average (Minimum~Maximum)	14.3 (6.2~27.8)

**Table 2:** Outcome according to the lateral pillar class.

Lateral Pillar Class	Good (Stulberg Class I or II)	Fair (Stulberg Class III)	Poor (Stulberg Class IV or V)
A	1	0	0
B	26	3	0
B/C, C	3	10	6

*p*-value < 0.001.

**Table 3:** Outcome according to age.

	Stulberg Class I or II	Stulberg Class III, IV, or V	*p*-Value
Group I (age < 4 years)	14	11	0.162
Group II (age ≥ 4 years)	18	6	

Group I (*n* = 25), group II (*n* = 24).

**Table 4:** Durations of the initial and fragmentation stages in each outcome group.

	Stulberg Class I or II	Stulberg Class III, IV, or V	*p*-Value
Initial	4.1 (0.7~9.8)	6.2 (2.6~13.5)	0.009
Fragmentation	5.9 (1.0~16.0)	11.9 (4.2~23.6)	<0.001

Mean durations of the initial and fragmentation stages were 5.0 and 8.2 months, respectively. *p*-value of the comparison between the durations of the initial and fragmentation stages <0.001.

**Table 5:** Comparison of the disease severity with the final outcome in previously reported studies.

	Lateral Pillar		Stulberg		
	A or B	B/C or C	I or II	III	IV or V
Rosenfeld et al. [6]	115 (61.2)	73 (38.8)	152 (80.9)	17 (9.0)	19 (10.1)
Gent et al. [5]	39 (56.5)	30 (43.5)	45 (65)	14 (20)	10 (15)
Nakamura et al. [7]	39 (34.2)	75 (65.8)	72 (63.1)	28 (25.6)	14 (12.3)
This study	30 (61)	19 (29)	30 (61)	13 (27)	6 (12)

## Data Availability

The data was not publicly available due to ethical reasons and patient privacy.
